# Social Representations of e-Mental Health Among the Actors of the Health Care System: Free-Association Study

**DOI:** 10.2196/25708

**Published:** 2021-05-27

**Authors:** Margot Morgiève, Pierre Mesdjian, Olivier Las Vergnas, Patrick Bury, Vincent Demassiet, Jean-Luc Roelandt, Déborah Sebbane

**Affiliations:** 1 WHO Collaborating Centre for Research and Training in Mental Health EPSM Lille Metropole Hellemmes France; 2 Cermes3 Centre de Recherche Médecine, Sciences, Santé Santé Mentale et Société Paris France; 3 Department of Emergency Psychiatry and Acute Care Lapeyronie Hospital CHU Montpellier Montpellier France; 4 University of Lille, EA 4354 Centre Interuniversitaire de Recherche en Education de Lille Lille France; 5 UFR Sciences Psychologiques & de l'Éducation University Paris-Nanterre Nanterre France; 6 Cleverside Courbevoie France; 7 Inserm Épidémiologie clinique, évaluation économique appliquées aux populations vulnérables UMR 1123 Paris France; 8 University Hospital of Lille Lille France

**Keywords:** e-mental health, social representations, free association task, psychiatry, mental health, mental health service users, technology, digital health

## Abstract

**Background:**

Electronic mental (e-mental) health offers an opportunity to overcome many challenges such as cost, accessibility, and the stigma associated with mental health, and most people with lived experiences of mental problems are in favor of using applications and websites to manage their mental health problems. However, the use of these new technologies remains weak in the area of mental health and psychiatry.

**Objective:**

This study aimed to characterize the social representations associated with e-mental health by all actors to implement new technologies in the best possible way in the health system.

**Methods:**

A free-association task method was used. The data were subjected to a lexicometric analysis to qualify and quantify words by analyzing their statistical distribution, using the ALCESTE method with the IRaMuTeQ software.

**Results:**

In order of frequency, the terms most frequently used to describe e-mental health in the whole corpus are: “care” (n=21), “internet” (n=21), “computing” (n=15), “health” (n=14), “information” (n=13), “patient” (n=12), and “tool” (n=12). The corpus of text is divided into 2 themes, with technological and computing terms on one side and medical and public health terms on the other. The largest family is focused on “care,” “advances,” “research,” “life,” “quality,” and “well-being,” which was significantly associated with users. The nursing group used very medical terms such as “treatment,” “diagnosis,” “psychiatry”,” and “patient” to define e-mental health.

**Conclusions:**

This study shows that there is a gap between the representations of users on e-mental health as a tool for improving their quality of life and those of health professionals (except nurses) that are more focused on the technological potential of these digital care tools. Developers, designers, clinicians, and users must be aware of the social representation of e-mental health conditions uses and intention of use. This understanding of everyone’s stakes will make it possible to redirect the development of tools to adapt them as much as possible to the needs and expectations of the actors of the mental health system.

## Introduction

### Context

Mental health care continues to face many challenges such as cost, accessibility, and the stigma associated with mental health. This results in inequalities and inadequacies in the treatment of many people with lived experiences of mental health problems [1]. The field of eHealth offers an opportunity to overcome these structural and personal barriers to seeking help [2]. Electronic mental health (or digital mental health) includes teleservice or telemedicine, interoperability repositories, shared medical records, mobile applications (mHealth), e-learning, online information searches and sharing, and others. Psychiatry, more than any other discipline, will be able to benefit from these new technologies. During the COVID-19 pandemic crisis, rapid virtualization demonstrated that clinicians, mental health service users, and health care systems were able to quickly adapt to telepsychiatry, overcoming previous obstacles including regulatory constraints, system inertia, and general resistance to telepsychiatry [3,4].

The use of technology is exponentially increasing in our society, especially the use of smartphones (more than 3.8 billion users worldwide) [5] to travel, communicate, work, manage one’s finances, or to have social relations. Recently, the World Health Organization (WHO) announced its intention to use “new opportunities, creativity, learning and technology [...] to ensure the health and well-being of everyone” [6]. The use of information and communication technologies (ICTs) in health care since the 2000s is already improving access to care by strengthening communication between health service users and providers and by making health systems and decisions more efficient and cost-effective [7]. Indeed, in addition to the provision of direct service delivery, eHealth enables people with lived experiences of mental health problems to access their shared medical records and receive medical advice and information directly on their computers, tablets, or smartphones.

However, the use of these new technologies in the area of health and mental health remains weak. In France, only 6% of the population have already experienced a teleconsultation, and 9% of health care professionals have already done (at least) a teleconsultation with one of their patients [8]. Also, nearly two-thirds of the French population report they are not ready to use connected objects in the future in the health care field [9]. On the contrary, people with lived experiences of mental health problems are more and more connected, and most are in favor of using applications and websites to manage their mental health problems [10].

This study explored the social representations of e-mental health with the actors of the mental health system with the hypothesis that these social representations can help to understand and characterize the intentions of use.

### Social Representations in eHealth

Several questionnaires were created in order to get an understanding of the barriers to the use of new technology in general. Those Technology Acceptance Models (TAMs), created in the 1980s, were used to better target the eHealth expectations of users [11] and professionals [12] based on 2 main questions: “Is this new technology useful for me” and “Is this technology easy to use?” Some researchers [13,14] have highlighted the need to broaden this questioning to include environmental factors of individuals, including the social influence between subjects but also between the tool and the subject. Indeed, this very logical-scientific approach to TAMs must be supplemented by a vision, certainly more subjective, but which directly questions social cognitions referring to the “object of e-mental health.” In order to understand the place of the individual in relation to this object in society and the socioeconomic power issues that emerge from it, it seems essential to question the mental image of e-mental health according to the beliefs and attitudes about it [15]. According to Jodelet [16], it is from this singular mental representation that a form of “knowledge is constructed, socially elaborated and shared, having a practical aim and contributing to the constitution of a reality common to a social group.” Thus, the social representation creates a link between the individual and the feeling of belonging to a group in society with the same interpretations and uses of e-mental health.

### Objective

This study aimed to characterize the social representations associated with e-mental health by all actors in order to implement new technologies in the best possible way within the health system.

## Methods

### Study Design

A qualitative study (EQUME) was conducted by the WHO Collaborating Centre of Lille (France) in order to assess the social representations and norms of 10 typologies of actors involved in the health care system. These 10 categories were chosen in order to have access to different professional groups with different references and practices (general practitioners, psychiatrists, social workers, psychologists, occupational therapists, and nurses); to service users and family carers; to user representatives, who have a discourse significantly different from the users; and to the general public. Participants were recruited through announcements to various professional networks, peer support groups, user and carer representatives (mainly posted on their respective websites), and by word of mouth. The inclusion criteria were as follows: (1) belong to one of these 10 categories of actors, (2) speak the French language, (3) be of legal age, (4) agree to participate in this study. There were no other criteria for noninclusion. The data were collected during focus groups (moderated by a social sciences researcher and an assistant psychiatric moderator), which took place in 2 French cities.

The first part of the study, based on data collected in focus groups, revealed a heterogeneous and unstable definition of e-mental health with regard to the different groups of actors concerned as well as within each group [17]. The second part of the study, presented here, is based on the free-association task method.

Each focus group was initiated with a sociodemographic questionnaire collecting the following variables: age, gender, and profession. Each group was then asked to complete a self-reported familiarity scale ranging from 0 to 10 with e-mental health devices. Finally, a free-association task — detailed in subsequent sections — was conducted to collect words related to e-mental health.

The EQUME study was the subject of a declaration of compliance with reference methodology at the French National Agency for Medicines and Health Products Safety (N°2040798 v 0, March 3, 2017). All participants were asked to sign a consent form.

### Procedures and Methods

The free-association method was chosen to study the social representations of e-mental health. This method is based on a question of evocation (or word associations) with the following written instructions: “Quote three words related to ‘e-mental health,’ then three more words related to these words” (see Multimedia Appendix 1). This exercise will result in having 3 words at the first level, then 9 words at the second level as each word from the first level will be associated with 3 other words. This makes a total of 12 words or expressions per participant. The free-association method is a classic tool in studies on social representations [18-20]. It calls upon the latent content of representation [15,21] opening a path to the semantic field of the social object studied through the spontaneity and projective dimension of the method of free associations [15]. According to Abric [15], social representation is composed of a content (eg, information, opinions, beliefs, attitudes) and a structure. The structure consists of a central system (or central core) and a peripheral system, each of which is composed of the beliefs of the same name. The central elements have “evidential status” and help to “provide a framework for interpreting and categorizing new information” [15]. The peripheral system links the central core of the representation to the reality of the moment for individuals. For example, if we consider “knowledge acquisition” as the central core of the object “study,” for some, “the library” will be a peripheral element, while for others, “the scholarship” will be an entirely different one (considering that knowledge acquisition would allow one to obtain a scholarship related to further study).

### Data Analysis

We used several types of text data analysis (TDA) in this study. TDA corresponds to a set of methods that aim to analyze the information contained in a text. Two of the authors (OLV and PB) who specialize in the statistical analysis of textual data conducted the technical analyses, guided by a social sciences researcher (MM) and mental health clinicians (PM and DS). They use categories to qualify elements of the text and quantify them by analyzing their statistical distribution.

### Lexical Analysis

The data were subjected to a lexical or lexicometric analysis: the ALCESTE method. It was developed by Reinert [22] on the basis of the work by Benzécri and the textual statistics of Lebart and Salem. We used the IRaMuTeQ software, which is open source, is free, uses the R language [23], and was developed by Ratinaud and Dejean.

Text segments have been created from each “level 1 word” and the three “level 2 words” associated with them (equivalent to a branch of the tree structure of the free-association diagram, see Multimedia Appendix 1). This makes 3 sentences or text segments called B1, B2, B3 (in the order of the word branches quoted from left to right on the diagram) per participant. In order to identify groups of words often together in these text segments, the analysis performed is mainly a Hierarchical Descending Classification (HDC). The software builds a tree structure, and a classification is proposed grouping the words most often used together in the same sentences or segments.

Still using the IraMuTeQ software, we obtained a visualization of the relations between the word clusters and the variables studied (age, sex, familiarity with e-mental health, categories of actors, order of text segments [ie, B1, B2, and B3]) with the corresponding chi-squared value (*P*=.05). We defined the significance threshold at 5%. Based on correspondence factor analysis (CFA) applied to the center of the clusters, this visualization provides pairs of images that can be stacked together. One of the images represents the relative proximity of words, and the other represents the types of text segments concerned, around the centers of these lexicons. The central words are the most common, and the distance from the center indicates the specificity of one or the other word. The axes mathematically maximize the visibility of specificities, but their orientation on the page (top/bottom and right/left) is arbitrary.

### Thematic or Categorical Analysis

To deepen the links that exist between the different terms, we used a graph representation tool (Neovis) in order to visually understand the different word associations. For this purpose, 3 researchers independently classified the 180 terms in level 1 into 24 categories.

On a technical level, the graphs were built from the Excel file resulting from the encoding of the responses that we injected into a dedicated database (neo4j) using a python script. An HTML page connecting to this database was then built; its role was to retrieve the relationship of interest and represent it using Neovis.

## Results

### Baseline Assessment

The sample comprised a total of 70 people (37 women and 33 men) between 24 and 77 years old (average age of 44 years; Table 1). They correspond to 10 categories of actors: general practitioners, psychiatrists, user representatives, general public, family carers, social workers, psychologists, service users, occupational therapists, and nurses.

Self-reported familiarity with e-mental health ranged from 0 to 9/10 but was on average, very low for all groups. The occupational therapists report the lowest level of familiarity (1.1), while general practitioners report the highest level of familiarity (4.5).

Responses to the free-association questionnaire had 167 words missing for 828 possible answers (20.2%). The user group had the highest rate of missing words (46%), as it was the group with the most participants.

**Table 1:** Participants’ characteristics and self-assessment of eHealth knowledge.

Categories of actors	Participants, n				Age of participants (years), mean (range)		Knowledge of e-mental health tools, mean (range)
	Men	Women	Total				
General practitioners	4	1	5	48.4 (40-59)		4.5 (3-5)	
Psychiatrists	3	2	5	43.6 (25-62)		3.2 (0-8.5)	
User representatives	2	1	3	54.3 (29-77)		3.3 (1-6)	
General public	0	6	6	38.5 (29-53)		3.2 (1-7)	
Family carers	6	3	9	62.2 (48-74)		1.8 (0-4)	
Social workers	0	5	5	43.2 (29-57)		1.6 (0-5)	
Psychologists	1	6	7	35.7 (25-59)		1.7 (0-5)	
Service users	11	1	12	42 (30-59)		3.7 (0-9)	
Occupational therapists	2	7	9	38.4 (24-56)		1.1 (0-4)	
Nurses	4	5	9	36.7 (25-48)		2.6 (0-6)	
Total group	33	37	70	44.3 (24-77)		2.2 (0-9)	

### Lexical Analysis

In order of frequency, the terms most frequently used to describe e-mental health in the whole corpus were “care” (n=21), “internet” (n=21), “computing” (n=15), “health” (n=14), “information” (n=13), “patient” (n=12), and “tool” (n=12).

The terms that were cited several times together (co-occurrences) throughout the corpus were “internet” and “information” (n=6), “internet” and “computer” (n=6), “activity” and “workshop” (n=5), “activity” and “care” (n=5), “hope” and “activity” (n=5), “carefree” and “activity” (n=5), and “will” and “activity” (n=5).

**Figure 1:**
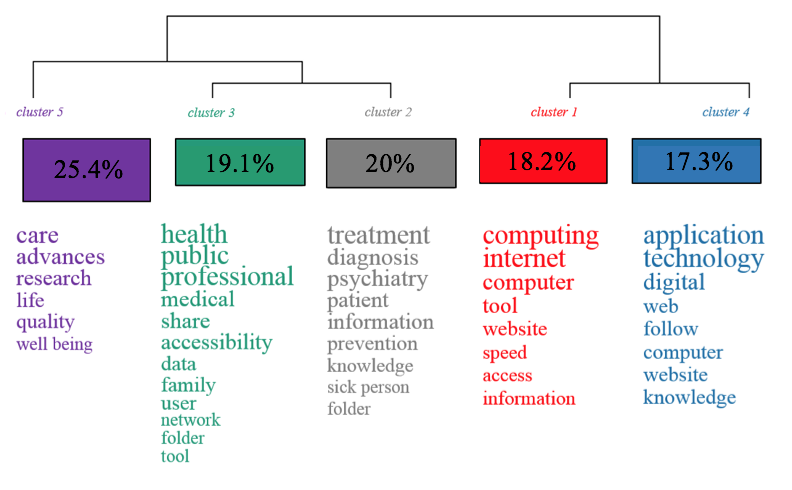
Classification of words used according to their frequency of co-citation within the same text segment by all participants in the 5 clusters, with *P*<.05 for all words.

Analysis of the similarities and differences between the terms used by the participants (Figure 1) shows 5 clusters of word characteristics in the main themes addressed. The percentages represent the number of times the words are cited together throughout the corpus.

The corpus of text is divided in 2, with technological and computing terms on one side and medical and public health terms on the other.

As for the medico-social terms, the largest family (cluster 5) is focused on “care,” “advances,” “research,” “life,” “quality,” and “well-being.” It is related to 2 families (clusters 2 and 3), which also include health-related terms but differ from them by more general terms. These 2 other clusters are distinguished by more specific terms related to psychiatry and preventive medicine (eg, “psychiatry,” “diagnosis,” “prevention,” “information”) and by access terms related to public health and the direct environment of the user of the health system (eg, “health,” “public,” “share,” “user,” “family”).

**Figure 2:**
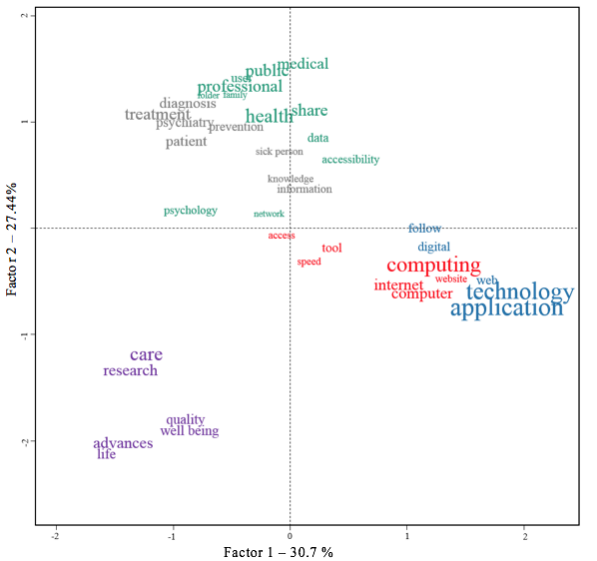
Correspondence factor analysis of the free-word association about e-mental health.

**Figure 3:**
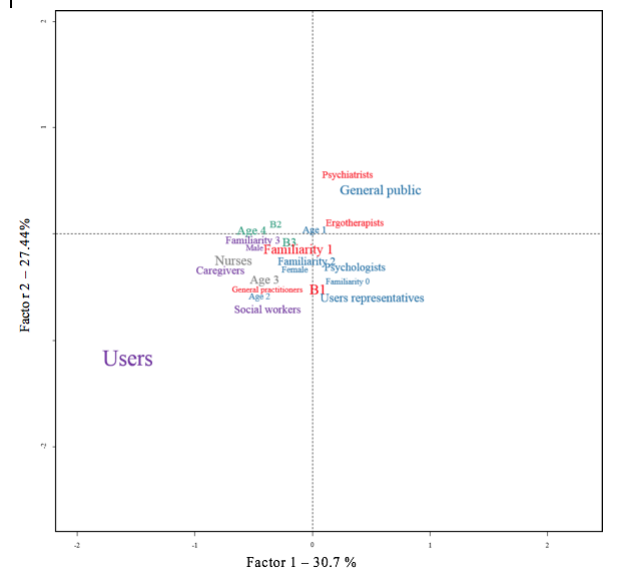
Correspondence factor analysis with the different variables studied. B1: text segment 1; B2: text segment 2, B3; text segment 3; Age 1: 20-30 years old; Age 2: 31-40 years old; Age 3: 41-60 years old; Age 4: ≥61 years old; Familiarity 0: no answer; Familiarity 1: 0-2; Familiarity 2: 3-5; Familiarity 3: 6-9.

During the task of free association, we can see that the participants very frequently quoted in the first line of the response (χ^2^_1_=4.93, *P*=.02) terms associated with the lexical fields of technology and computer science (B1, Figure 2) overlapping with cluster 1 (Figure 3).

Participants over 61 years of age-related e-mental health to terms in the fields of “health,” “public,” “professional,” “medical,” “accessibility,” “family,” “user,” and “network” (χ^2^_1_=3.93, *P*=.04).

The CFA (Figures 2 and 3) shows that the group of users consist of those who use the terms focused on “care,” “progress,” “research,” “life,” “quality,” and “well-being” the most (χ^2^_1_=11.16, *P*<.001). It appears that this group of participants would make very little use of the other families of words, and almost none of them used terms related to technology or computing (clusters 1 and 4).

The nursing group used very medical terms such as “treatment,” “diagnosis,” “psychiatry,” and “patient” to define e-mental health (χ^2^_1_=4.8, *P*=.02). They also used more global words focusing on “quality,” “care,” “progress,” and “well-being” as well as “users.” They did not associate e-mental health at all with the terms in the public health–oriented family (cluster 3).

The general public group associated terms such as “application,” “technology,” “digital,” “web,” “monitoring,” “computer,” “site,” and “knowledge” with e-mental health (χ^2^_1_=4.63, *P*=.03), as did the group of user representatives (χ^2^_1_=3.11, *P*=.07).

The general public, psychiatrists, occupational therapists, user representatives, and general practitioners used very little or no medico-social vocabulary (from clusters 2, 3, and 5) within their representations of e-mental health. These groups are more likely to use terms focused on computing and technology.

### Semantic Analysis

**Figure 4:**
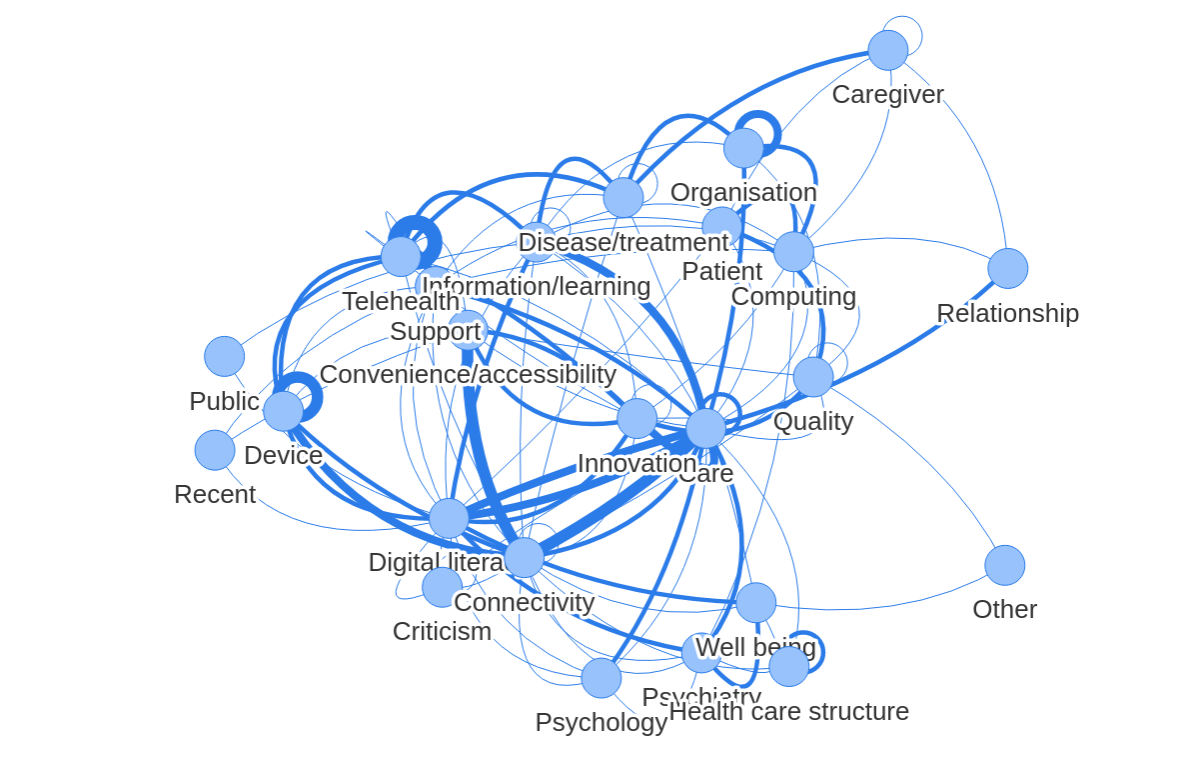
Links between ad hoc categories of level 1 words. The stroke thickness and node distances represent the frequencies of word co-occurrences between categories.

The 24 ad hoc categories constituted by the investigators and based on level 1 terms are illustrated in Figure 4. This graph summarizes the main corpus by lexical fields. It introduces a dynamic dimension by adding links between the different categories, which can be compared to more or less “stretched springs” depending on the number of relationships between groups of words.

As shown in the figure, it is possible to notice that the central place of the category “care” has a link with almost all the other categories. The terms constituting this category are therefore related at least once to the words used in the other categories.

The other graphs that were made according to the different variables (age, gender, familiarity with e-mental health, except groups of participants) show a core of close relationships between the categories “care,” “connectivity,” “pathology/treatment,” “device,” “telehealth,” “computing,” “information/training,” “digital literacy,” “practicality/accessibility,” and “innovation.” There was no clear structural difference in the graph (Figure 4). It is possible to observe differences between the groups of participants depicted in the graphs; however, it is not possible to provide a clear conclusion because of the low number of participants per group.

## Discussion

### Main Findings

The scores of the self-reported familiarity scales are generally below average and are opposed to the richness of the words and lexical fields mobilized by the participants during the task of free association. This highlights a necessary distinction between daily digital use and access to digital health literacy that is controlled [24]. It is the responsibility of the state to set up an education system at school that allows future e-citizens to know how to use these tools in an informed way and to manage their digital identities and a digital infrastructure in order to avoid the “digital divide” as well as digital health illiteracy [7].

In the main corpus, a homogeneous and frequent vocabulary field relating to health care and ICTs (Figure 4) allows us to formulate the hypothesis of the centrality of these lexical fields illustrating the social representations of e-mental health of the participants. The absence of terms with positive or negative valences is to be noted. In addition, a very consensual and materialistic definition characterizes the central system of social representation. e-Mental health is considered a new technological, computerized, and medical tool that would be able to offer a diagnosis or treatment to people with a lived experience of mental health problems. These tools are at the service of information and training. Data from the free-association task suggest a relative openness or at least a lack of aversion to the mental health of participants. The subsequent focus group discussions also point in this direction, but nevertheless highlighted fears linked to “dehumanization” or the replacement of humans by technological tools [17]. The peripheral elements are linked to the structural and organizational dimensions of e-mental health (ie, “structure of care,” “organization”).

The group of service users of the mental health system is clearly distinguished by a specific vocabulary. It differs from the words most found in the main corpus but also from the other groups of participants. These discrepancies evoke the nuance between users’ expectations of improving their quality of life in the first place and that of health professionals (except nurses), which focused more on the potential of new digital tools to perform repetitive tasks for them, allowing them to refocus their practice on what makes (clinical) sense.

“Psychiatry” and “psychology” are also peripheral elements of the representation of e-mental health. While psychiatry has established itself as the “normal practice” that has regulated the conception of disorders and their treatments for many years, it may seem “natural” that it now extends its jurisdiction to the field of mental health. This extension could thus announce the renewal of psychiatric practices, as well as their social role. Current frameworks guiding clinical practice in psychiatry and psychology are limited because they do not address the complex reality of people with lived experiences of mental health problems. They project on them a predefined reading grid and neglect the dynamic interaction between their real, lived experiences, which are inextricably linked to social, psychological, and biological contexts [25]. The mental health care system thus does not consider the fundamental realities of people with lived experiences of mental health problems in their daily life. We therefore urgently need new paradigms of clinical practice to effectively treat these people in vivo, in which what matters the most for them — loss of meaning, impoverishment, social isolation, and/or disability associated with symptoms — is also what matters the most to clinicians [26,27]. However, new digital tools can precisely enable people to be observed and treated in vivo, by integrating a stream of ecological and multidimensional data. These developments require theorizing methodological approaches to guide the design of new digital tools adapted to the challenges of a digital clinic.

This integration of digital tools in the daily practice can thus become part of a “professional project” in order to gain status and expand territories [27].

“Well-being” is also a peripheral element associated with “care” for users specifically. This representation illustrates the process of gradually extending psychiatry to “mental health” and even happiness since the 1980s. This extension is based on the redefinition of health by the WHO, no longer as the absence of disease but as “complete physical, mental and social well-being.” “Mental health” has become ubiquitous in public health discourse and more broadly throughout the social landscape since the early 2000s. Many actors in the field see it as a form of injunction to happiness and well-being beyond the scope of psychiatrists’ interventions. This presence of “well-being” in the discourse of users can also be explained by the fact that the current technological tools are not necessarily medical tools but common objects (eg, connected watches, actimetry bracelets, smartphone applications) that have been designed according to ways of thinking about the world from fields other than psychiatry, in particular the well-being and quantification of oneself.

“Relationship” is also one of the peripheral representations associated with e-mental health. New technologies are changing the relationships between caregivers and people with lived experiences of mental health problems, enabling new forms of digital intimacy thanks to a new form of continuity of care. According to Fairhurst and May [28], abstract medical knowledge (“knowing the patient”) can thus be supplemented and enriched by personal exchanges that can help the clinician to “know about the patient.” The most recent example comes from the COVID-19 health care crisis during which telepsychiatry allowed the maintenance of social links through digital health despite the need for physical distancing [29]. Although technology has enabled the maintenance of caregivers’ and patients’ “connection,” experts recommend the complementary use of telepsychiatry with face-to-face interviewing [3,29]. It is a question of finding the balance of these new hybrid relationship modalities within the patient-caregiver-technology triad. Foucault et al [30] raised the following question: “Is there a virtual saturation point at which the benefits of a virtual relationship diminish, or patients demand more face-to-face interactions?” The relationship between service users and professionals seems to be evolving towards a rebalancing of each other’s roles and is being profoundly transformed under the effect of new technologies; the e-citizen user is thus becoming an informed actor of his or her health, expert, and partner in an increasingly digitalized ecosystem.

Although the processes of “autonomy” and empowerment are recommended by public health authorities and that these terms are increasingly present in the contemporary discourses of patient and service user associations as well as more widely disseminated in society, it is surprising that they are totally absent from the task of free association. However, the discursively configured involvement of patients in their care through technology is a matter of debate: advocated by some as a means of horizontalizing the caregiver-patient relationships and contested by others as a social injunction and the sign of the expression of a Foucauldian bio-power [30]. The promising discourse of digital health policy positions citizens as objects of political intervention but neglect the many social, political, cultural, and economic inequalities that specifically prevent engagement in digital health [31].

Similarly, “data” is absent from free associations. Participants thus did not seem to question the place of their own data in the mental health ecosystem or to be concerned about the use that private lobbies can make of it. This absence of “data” from the discourses of all the typologies of actors can be interpreted at different levels. Users of the health system may seem to “not care” about the confidentiality and security of their data. There might be a few reasons for that: They may trust the e-mental health ecosystem, it may seem that the benefit-risk balance makes it preferable for them to use these digital tools, or they might not have mastered the issues related to the use and circulation of their data. This could be explained by the difficult acquisition of health literacy for users of the health care system as well as for professionals, not because of lack of interest but rather by the complexity of the health ecosystem (ie, the lack of resources and reliable evaluation of medical information on the internet). To use the concept by Petersen et al [32], “cartographies of trust” has now become extremely complex and follows tortuous and emotionally charged paths that require navigating between online and offline resources. Health literacy is evolving; it requires medical, informational, and more recently, digital skills [24]. Considered increasingly civic and social [33], it is now part of the community with, on the one hand, the need for an awareness of “self-concern” at the individual level [31] and, on the other hand, the need for optimal organization of the health ecosystem managed by the guardianships.

As Henwood and Marent [31] rightly pointed out, at the level of the individual, the ways people “make sense with numbers” and numbers “make sense of people” interact so finely that it is extremely complex to determine towards or tilt the balance between freedom and power, determining and being determined, acting and being acted upon; in such a way, it is urgent to expand our sociological imaginations of the “reflexive” patient or citizen.

### Limitations

The aim of this study was to “photograph” the social representations of e-mental health from the different typologies of actors in the health care system. The speed of development of new devices implies new uses will likely have a retroactive impact on users’ representations, making it difficult to capture these constantly evolving representations. Also, one of the main limitations of our work is related to the small number of participants present in each group. Our material has a certain number of nonresponses to the task of free association without the possibility of exchange with participants. More qualitative studies, using narrative content, interviews, focus groups, and field observation methodologies, are needed to further explore the social representations of e-mental health among different actors.

### Conclusions

The rise of e-mental health in our health systems is both a challenge and an opportunity for mental health. This study showed that the social representations of e-mental health differ according to the social group to which participants belong. It conditions an intention of use that developers, designers, clinicians, and users must be aware of. This better understanding of everyone’s stakes will make it possible to redirect the development of tools and adapt them as well as possible to the needs and expectations of the actors of the mental health system. In this process of listening and horizontalization of the relationships between actors, the aim was to harmonize the contribution of digital tools and enable their appropriation by all users, as well as facilitating equal access to care by bridging the digital divide. In order to do so, the guardianships must ensure the deployment process of the tools. If all user citizens have to be concerned by these policies and if they are to remain committed to a better knowledge of themselves and their health, these reflections must be participatory and collaborative. In this sense, the improvement of the components relating to the training of actors through the acquisition of digital skills and the increase of literacy e-mental health is at the dawn of a successful implementation of digital mental health.

## Appendix

Multimedia Appendix 1 Free association task instructions.

## Abbreviations

CFA: correspondence factor analysis
HDC: Hierarchical Descending Classification
ICT: information and communication technology
TAMs: Technology Acceptance Models
TDA: text data analysis
WHO: World Health Organization
