# Supplementary figures and images for: Social Representations of e-Mental Health Among the Actors of the Health Care System: Free-Association Study
Source: JMIR Ment Health. 2021 May 27;8(5):e25708. doi: 10.2196/25708 (PMC8193480; doi:10.2196/25708)

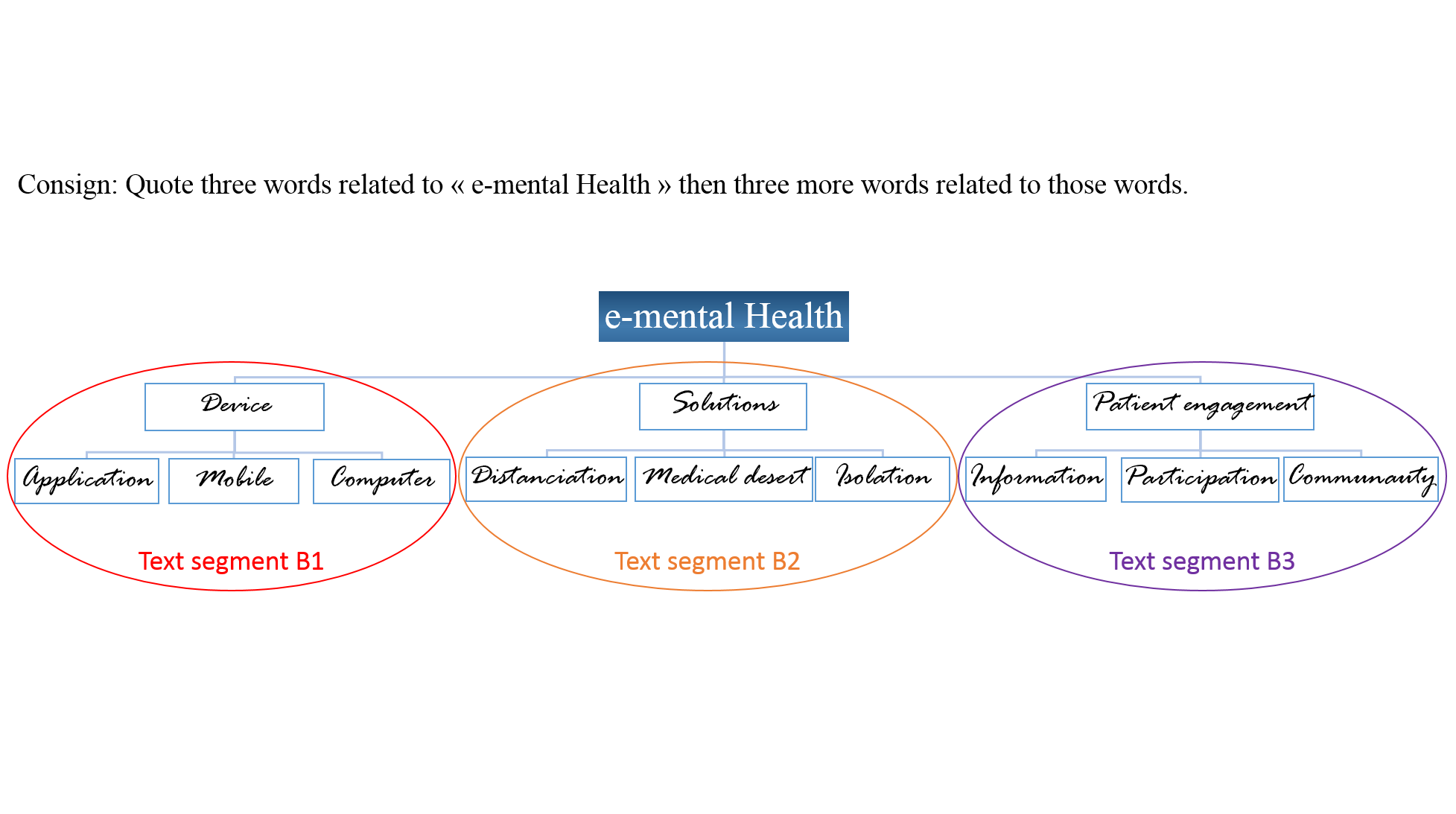

Supplement: Multimedia Appendix 1 [file mental_v8i5e25708_app1.png]
